# Impact of genotypes, environmental stresses, and genotype by environment interactions on growth and yield of quinoa at flowering stage

**DOI:** 10.1371/journal.pone.0331652

**Published:** 2025-09-02

**Authors:** Van Loc Nguyen, Trung Hieu Do, Thi Hong Nhung Phan, Viet Long Nguyen, Duc Ha Chu, Daniel Bertero, Néstor Curti, Viet Ton Ta, Peter C. McKeown, Charles Spillane

**Affiliations:** 1 Faculty of Agronomy, Vietnam National University of Agriculture, Hanoi, Vietnam; 2 Student at Faculty of Agronomy, Vietnam National University of Agriculture, Hanoi, Vietnam; 3 Faculty of Agricultural Technology, University of Engineering and Technology, Vietnam National University Hanoi, Hanoi, Vietnam; 4 Depto. de Producción Vegetal, Facultad de Agronomía, Universidad de Buenos Aires and IFEVA-Conicet, Buenos Aires, Argentina; 5 Escuela de Agronomía, National University of Salta, Salta, Argentina; 6 Mathematical Modeling Laboratory, Kyushu University, Fukuoka, Japan; 7 Agriculture, Food Systems & Bioeconomy Research Centre, Ryan Institute, University of Galway, Galway, Ireland; Brigham Young University, UNITED STATES OF AMERICA

## Abstract

Flowering is a critical growth stage of quinoa (*Chenopodium quinoa* Willd.), with a strong influence on growth and grain yield. To understand factors affecting such flowering stage effects, we measure the differential effects of genotype (G), environmental stress (E), and genotype by environment interaction (G × E) on quinoa growth and yield-related traits during the flowering stage. A semi-controlled pot experiment was conducted in a greenhouse using a Randomized Complete Block Design (RCBD) with five replications. Five quinoa genotypes (Q1, Cahuil, G18, Isluga, and Q3) were evaluated under four climate-related stress vs non-stress treatment conditions: control (E1), waterlogging (E2), salinity (E3), and drought (E4). Morphological and yield traits, including plant height, number of tillers and leaves, leaf area, soil plant analysis development (SPAD) values, fresh and dry biomass, panicle length, 1000-grain weight, and individual grain yield were measured. There were significant effects of G, E, and G × E interaction on all measured traits, indicating considerable variation in genotype adaptability to abiotic stresses. The order of stress severity was E2 > E4 > E3 > E1, with waterlogging causing the most substantial reductions across growth and yield traits. The AMMI analysis highlighted strong genotype-specific responses across environments. Our findings provide insights into how quinoa responds to environmental stresses, supporting the development of research strategies and and irrigation management for quinoa under climate change related stresses.

## Introduction

Quinoa (*Chenopodium quinoa*) is a small grain crop whose center of origin is in the Andean highlands of South America [1]. Quinoa has long been cultivated for its nutritional benefits [2] and its capacity to grow under a wide range of environmental conditions [3]. With its high protein levels and complete amino acid profile, quinoa has become increasingly important in global agricultural systems, particularly for enhancing food and nutritional security [4]. The crop’s expansion beyond its center of origin and traditional growing regions reflects rising interest in its agronomic potential, nutritional and economic value. However, quinoa’s productivity is constrained by abiotic stress factors, such as drought, salinity, and waterlogging, which can be increased in frequency and intensity by climate change [4]. While a number of studies have examined the effects of abiotic stresses on quinoa during the germination and seedling stages, a notable gap remains in understanding how abiotic stresses impacts the plant during the flowering stage. The flowering phase is a particularly sensitive developmental stage, because stress at this stage can severely disrupt floral development, reduce seed set, and ultimately decrease grain yield per plant. Hence, understanding the effects of environmental stresses during flowering stage of quinoa is important to select genotypes that can perform well across multiple locations under abiotic stresses. The identification of the best performing genotypes across different stresses is critical for selection of genotypes for climate-resilient crop production and breeding.

Abiotic stresses such as drought, salinity, and waterlogging significantly hinder plant growth and productivity, especially during the flowering stage, a critical phase for reproductive success and yield formation [5]. All three abiotic stresses can worsen in severity and frequency under climate change. Drought stress during the flowering period reduces water availability, leading to stomatal closure, decreased photosynthetic activity, and disruption of floral organ development, which ultimately reduces pollen viability, pollination efficiency, fertilization, and seed set, while increasing flower and seed abortion, with concomitant effects on yield [6]. For example, in soybean (*Glycine max*), flowering-stage drought decreases photosynthesis, pollen fertility, and pod formation [7]; in sorghum (*Sorghum bicolor*), it limits flowering, panicle exertion, and seed filling [8]; in chickpea (*Cicer arietinum),* it disrupts assimilate partitioning, reducing flower and pod numbers and final seed yield [9]; and in cotton (*Gossypium hirsutum*), it causes decreased flower and boll yield due to reduced carbohydrate supply and hormonal imbalance [10].

Climate change leads to rises in sea levels and changing rainfall patterns, both of which can increase saltwater intrusion into freshwater, thereby increasing salinity in agricultural lands. Salinity stress during crop flowering affects reproductive development by inducing ionic toxicity and osmotic imbalance, which impair water and nutrient uptake and lead to malformed flowers, delayed anthesis, early senescence, and reduced seed development and yield [11]. In rice (*Oryza sativa*), high salinity reduces spikelet fertility and grain number [12]; in tomato (*Solanum lycopersicum*), it disrupts potassium and calcium balance, inhibiting pollen tube growth and causing floral drop [13]. In addition, waterlogging depletes oxygen in the root zone, compromises root respiration, nitrogen assimilation, and hormonal regulation, thereby affecting floral differentiation and fruit set [14]. In wheat (*Triticum aestivum*), this reduces spikelet fertility and grain set [15], while in soybean it reduces nodule activity and causes flower abortion and yield decline [16]. Collectively, these three abiotic stresses (drought, salinity and waterlogging) during flowering delay floral initiation, reduce flower fertility, and ultimately decrease yield quantity and quality.

In quinoa (*Chenopodium quinoa* Willd.), there have been some studies of its tolerance to drought and salinity stress during flowering, both of which have been shown to reduce pollen viability, seed set, and yield. However, there is a lack of research focusing on the effects of waterlogging during the flowering stage [17]. In addition, most studies concentrate on terminal drought or salinity stress, often examining physiological or yield-related traits [18], while overlooking the reproductive sensitivity of quinoa to excess water. To date, no comprehensive comparative study has been conducted to systematically measure and compare the impacts of drought, salinity, and waterlogging on quinoa, during the flowering stage. This represents a research gap that limit understanding of quinoa’s resilience as a necessary basis for development of climate resilient cropping and breeding.

G × E describes how different genotypes respond differently to agro-environments [19], leading to trait variability betweeen environmental conditions [20]. Substantial G × E interactions have been documented in quinoa during multi-environment trials, which challenges quinoa breeding programs by complicating the selection process of superior genotypes that work well across environments (i.e., display broad adaptation). Multi-environment trials are critical for identifying quinoa genotype, displaying either broad or specific adapfation to environmental conditions [21]. In this study we investigated genotype-by-environment (G × E) interactions in quinoa at the flowering stage, with a focus on understanding how different genotypes respond to key environmental stresses such as waterlogging, drought, and salinity. Our findings can inform and enhance quinoa breeding programs to develop quinoa varieties with improved stress tolerance during this critical growth stage.

## Materials and methods

### Materials

The study employed five quinoa genotypes to investigate G × E interactions during the flowering stage. The genotypes (G-s) are Q1 (G1), Cahuil (G2), G18 (G3), Isluga (G4), and Q3 (G5). Q1 and Q3 were selected by Dr. Redouane Choukrallah at ICBA (UAE) based on their superior performance under hot environmental conditions [22]. G18 is a breeding line derived from crosses between *Chenopodium quinoa* and *Chenopodium berlandieri*, provided by Dr. Eric Jellen (Brigham Young University, USA). Cahuil is a germplasm originating from central Chile, whereas Isluga originates from northern Chile, near the border with Bolivia. These quinoa materials were provided through a project funded by the Vietnamese Ministry of Science and Technology (Grant No. HNQT/SPĐP/07.17) under the bilateral cooperation program between the Governments of Vietnam and Argentina for the evaluation of quinoa under different ecological conditions in Vietnam.

### Experimental design and treatments

The experiment was conducted under semi-controlled greenhouse conditions at the Faculty of Agronomy, Vietnam National University of Agriculture, Gia Lam District, Vietnam, with an average light intensity of 1150 μmol m^-2^ s^-1^. Seeds from each of the five quinoa genotypes were sown in plastic pots measuring 200 mm in bottom diameter, 300 mm in top diameter, and 200 mm in height, each filled with 5 kg of sieved sandy clay-loam paddy soil. Fertilizers were uniformly applied to each pot, providing 0.81 g of nitrogen (N), 0.54 g of phosphorus pentoxide (P_2_O_5_), and 0.54 g of potassium oxide (K₂O) per pot. At the 4–5 leaf stage, seedlings were thinned to three plants per pot and further reduced to two plants per pot at the 9–10 leaf stage. Soil moisture was maintained at field capacity (32% w/w) by replenishing with fresh water every two days. Beginning at 45 days after sowing, corresponding to the flowering stage, plants were subjected for 10 days to one of four environmental conditions (E-s), including control conditions (E1), waterlogging stress (E2) where pots were maintained with 20 mm of water above the soil surface, salinity stress (E3) involving the application of 500 ml of 200 mM NaCl (as the standard threshold for assessing salt tolerance in quinoa) [23] solution every three days, or drought stress (E4) where no water was supplied (soil moisture 16–18%). After the 10-day treatment period, half of the plants were sampled, while the remaining plants were returned to control conditions and grown until seed harvest for a second sampling. The experiment utilized a randomized complete block design, with each genotype × treatment combination replicated five times.

### Measurements

The morphological and yield-related traits assessed comprised plant height (PH), number of leaves (NoL), number of branches (NoB), leaf area (LA), SPAD chlorophyll index, fresh weight (FW), dry weight (DW), panicle length (LP), weight of 1000 grains (P1000), and individual grain yield (IY). PH, NoL, NoB, LA, SPAD value, FW, and DW were measured during the first sampling phase. PH was measured from the soil surface to the apex of the main stem. NoL and NoB were counted based on leaves exceeding 1 cm in length and the presence of axillary buds, respectively. LA was determined using a leaf area meter (Li-3100, Li-Cor Biosciences, USA). FW was recorded with an electronic balance (OHAUS PR4202, USA), and DW was obtained after drying the samples at 80 °C for three days in a drying oven (BINDER, USA) until a constant weight was achieved. During the second sampling, LP, P1000, and IY were measured. LP was assessed by measuring the primary panicle from its base to the tip. The P1000 and dry samples were weighed using an electronic balance (OHAUS AX-224, USA). IY was calculated at a standardized grain moisture content of 14%, which was determined using a portable moisture meter (PM650, Japan).

### Data analysis

Statistical analyses were performed using R software to assess the effects of genotype and environmental stress, and their interaction. An analysis of variance (ANOVA) was conducted to determine the significance of these factors on the measured traits. When significant effects were detected, mean comparisons were made using the Least Significant Difference (LSD) test at a 5% significance level. Hierarchical clustering and principal component analysis (PCA) were utilized to explore patterns and relationships among variables, employing the “factoextra” and “FactoMineR” packages in R version 4.1.3. To further investigate genotype performance across different environments, additive main effects and multiplicative interaction (AMMI) model and the genotype main effect plus genotype by environment interaction (GGE) biplot analyses were conducted using the “metan” package in R version 4.1.1.

## Results

### Effects of genotype, environment, and genotype × environment interaction on measured traits of quinoa

Quinoa is considered a promising crop for providing climate resilience due to its tolerance to various environmental stresses, yet the determinants of its response to abiotic stresses during the flowering stage have been little studied. We addressed this by growing five quinoa cultivars conditions in a randomized block design and subjecting them to three environmental stresses. We evaluated the effects of genotype, environment, and G × E in determining the cultivars responses using ANOVA on a suite of ten morphological and yield-related characteristics, including PH, NoL, NoB, LA, SPAD, FW, DW, LP, P1000, and IY (summarized in Table 1). The ANOVA revealed that the main effects of genotype and environment and their interactions were all significant (*p* < 0.05) across all measured traits, indicating that there is substantial variability attributable to genetic differences, environmental conditions, and their interactions.

**Table 1:** ANOVA for measured traits in quinoa.

Trait	*F-*value		
	*Genotype (G)*	*Environment (E)*	*G × E*
PH	7.200***	42.194***	4.168***
NoB	3.295***	0.806***	0.731***
NoL	4.751***	2.429‘^.^’	1.275
LA	183.2***	1652.7***	118.8***
SPAD	13.936***	25.703***	2.385***
FW	39.44***	211.00***	24.05***
DW	75.06***	123.81***	32.39***
LP	8.976***	7.663***	11.932***
P1000	1.746	4.042**	1.531
IY	21.27***	2.42‘^.^’	13.53***

Plant height (PH), number of leaves (NoL) and number of branches (NoB), leaf area (LA), fresh weight (FW), dry weight (DW), length of panicle (LP), weight of 1000 grains (P1000), individual grain yield (IY); The asterisks indicate significance at ***p < 0.001, **p < 0.01, *p < 0.05, ‘^.^’p < 0.1.

### Ranking of genotype and abiotic stress effects

To identify the factors with the strongest impact on stress response in these quinoa genotypes, we ranked the effect levels of genotypes on the measured traits, and did the same for effect levels of abiotic stresses (Tables 2–4). Among the genotypes, variations were observed in their performance across different traits (Table 3). For example, Genotype G1 (Q1) exhibited superior performance in traits such as NoL (~30 leaves per main stem) and NoB (~25 branches per main stem), while it recorded the lowest values in IY (4.45 g plant^-1^). The abiotic stresses imposed significant effects on the measured traits (Table 4). Compared to the E1, all three stress conditions (E2, E3, and E4) led to notable reductions in multiple traits (Table 2). Among them, E4 caused the most substantial decreases, with values dropping to 75.3% for PH, 40.9% LA, and 45.9% for FW; with representing reductions of 24.7%, 59.1%, and 54.1%, respectively. E3 also had significant effects, reducing PH by 21.0%, LA by 50.8%, and FW by 38.6%. In contrast, while E2 had less impact on these three traits, it showed the most severe effects on other characteristics, including NoL (11.2%), NoB (8.4%), and SPAD value (23.4%), as well as notable reductions in LP (11.3%), P1000 (10.0%), and IY (9.7%).

**Table 2:** Mean of measured traits in quinoa.

Environment (E)	Genotype (G)	PH	NoL	NoB	SPAD	LA	FW	DW	LP	P1000	IY
E1	G1	100.4	32.6	28.8	60.84	479.78	36.51	11.07	13.76	3.68	4.29
	G2	94.0	27.0	22.4	54.28	698.69	54.08	9.32	16.96	3.45	10.29
	G3	109.4	25.4	20.6	53.86	440.02	43.52	10.46	12.22	3.36	5.36
	G4	105.2	24.6	19.2	59.10	528.51	42.08	9.22	11.42	3.24	5.61
	G5	104.0	29.0	22.0	60.90	848.64	55.97	8.66	13.22	3.30	8.01
***Average***		***102.6***	***27.7***	***22.6***	***57.80***	***599.13***	***46.43***	***9.75***	***13.52***	***3.41***	***6.71***
E2	G1	79.4	24.8	20.8	47.74	262.10	17.72	6.65	11.80	3.08	4.28
	G2	100.4	27.6	23.4	49.00	459.06	36.98	6.33	12.04	3.26	6.63
	G3	88.2	23.6	19.6	37.32	245.56	42.00	7.48	10.96	3.44	4.86
	G4	97.4	23.0	19.6	36.16	306.30	23.40	10.22	12.70	2.59	6.23
	G5	78.2	23.8	20.0	51.24	348.49	50.04	7.77	12.44	2.97	8.29
***Average***		***88.7***	***24.6***	***20.7***	***44.29***	***324.30***	***34.03***	***7.69***	***11.99***	***3.07***	***6.06***
E3	G1	88.2	29.6	24.0	54.46	368.41	35.41	7.32	9.98	3.14	3.77
	G2	80.6	24.8	21.0	38.74	334.10	35.09	9.56	12.84	3.25	5.95
	G3	91.8	27.6	22.2	33.98	304.01	22.66	10.05	15.32	3.46	8.31
	G4	72.0	24.8	19.4	43.92	249.50	25.18	10.92	11.80	3.39	6.48
	G5	72.8	25.8	21.6	53.62	219.03	24.21	6.26	12.72	3.39	5.92
***Average***		***81.1***	***26.5***	***21.6***	***44.94***	***295.01***	***28.51***	***8.82***	***12.53***	***3.33***	***6.08***
E4	G1	74.0	31.0	24.2	51.16	245.67	20.25	5.34	15.62	3.03	6.09
	G2	86.6	27.2	20.4	52.30	296.85	27.95	8.41	13.78	3.41	6.02
	G3	87.3	25.2	20.5	42.38	260.66	24.31	7.55	12.96	3.26	7.02
	G4	75.2	24.4	20.2	47.50	208.06	17.67	8.60	11.02	3.23	7.03
	G5	63.4	21.0	21.4	52.22	216.79	16.28	6.33	11.72	3.06	5.16
***Average***		***77.3***	***25.8***	***21.3***	***49.11***	***245.61***	***21.29***	***7.25***	***13.02***	***3.20***	***6.26***
***LSD***_***0.05***_ ***for G***		***4.5***	***2.7***	***2.8***	***3.86***	***12.32***	***2.33***	***0.32***	***0.73***	***0.23***	***0.61***
***LSD***_***0.05***_ ***for E***		***4.9***	***2.4***	***2.5***	***3.45***	***11.02***	***2.08***	***0.29***	***0.65***	***0.21***	***0.55***
***LSD***_***0.05***_ ***for G × E***		***11.0***	***5.4***	***5.6***	***7.72***	***24.65***	***4.66***	***0.65***	***1.46***	***0.46***	***1.23***

Plant height (PH), number of leaves (NoL) and number of branches (NoB), leaf area (LA), fresh weight (FW), dry weight (DW), length of panicle (LP), weight of 1000 grains (P1000), individual grain yield (IY).

**Table 3:** Ranking of the effect levels of genotypes on plant height (PH), number of leaves (NoL) and number of branches (NoB), leaf area (LA), fresh weight (FW), dry weight (DW), length of panicle (LP), weight of 1000 grains (P1000), and individual grain yield (IY) of the quinoa plant under environmental stresses.

Trait	Ranking of genotypes				
PH	G3^a^	G2^ab^	G4^b^	G1^b^	G5^c^
NoB	G1^a^	G2^ab^	G5^b^	G3^b^	G4^b^
NoL	G1^a^	G2^b^	G3^b^	G5^b^	G4^b^
LA	G2^a^	G5^b^	G1^c^	G4^d^	G3^e^
SPAD	G5^a^	G1^a^	G2^b^	G4^b^	G3^c^
FW	G2^a^	G5^a^	G3^b^	G1^c^	G4^c^
DRY	G4^a^	G3^b^	G2^c^	G1^d^	G5^e^
LP	G2^a^	G1^b^	G3^b^	G5^b^	G4^c^
P1000	G3^a^	G2^ab^	G1^ab^	G5^ab^	G4^b^
IY	G2^a^	G5^ab^	G3^b^	G4^b^	G1^c^

Different lowercase letters within rows represent statistically significant differences (Least significant Difference test, p < 0.05) where a is the largest value. G1, G2, G3, G4, and G5 represent the Q1, Cahuil, G18, Isluga, and Q3 genotypes, respectively.

**Table 4:** Ranking of the relative strength of environmental stresses on quinoa.

Trait	Ranking of environmental effects			
PH	E1^a^	E2^b^	E3^c^	E4^c^
NoB	E1^a^	E3^a^	E4^a^	E2^a^
NoL	E1^a^	E3^ab^	E4^ab^	E2^b^
LA	E1^a^	E2^b^	E3^c^	E4^d^
SPAD	E1^a^	E4^b^	E3^c^	E2^c^
FW	E1^a^	E2^b^	E3^c^	E4^d^
DW	E1^a^	E3^b^	E2^c^	E4^d^
LP	E1^a^	E4^ab^	E3^bc^	E2^c^
P1000	E1^a^	E3^ab^	E4^bc^	E2^c^
IY	E1^a^	E4^ab^	E3^b^	E2^b^

Traits are plant height (PH), number of leaves (NoL) and number of branches (NoB), leaf area (LA), fresh weight (FW), dry weight (DW), length of panicle (LP), weight of 1000 grains (P1000), and individual grain yield (IY). Different lowercase letters within rows represent statistically significant differences (Least Significant Difference test, p < 0.05) where a is the largest value. E1, E2, E3, and E4 represent four environmental stresses: control, waterlogging, salinity, and drought.

### Genotype × environment interaction and stability analysis

As it is likely that quinoa germplasm contains under-evaluated genetic variation for its environmental responses, we also ranked the effect levels of G × E interactions on the growth and yield of quinoa at the flowering stage (Table 5). Significant G × E interactions were detected for all traits, indicating that the performance of genotypes varied widely across different environmental conditions. This variability underscores the importance of evaluating genotypes under multiple environments to identify stable performers.

**Table 5:** Ranking of the relative strength effects of genotype by environment (G × E) interactions.

PH	NoB	NoL	LA	SPAD	FW	DW	LP	P1000	IY
G3E1^a^	G1E1^a^	G1E1^a^	G5E1^a^	G5E1a	G5E1^a^	G1E1^a^	G2E1^a^	G1E1^a^	G2E1^a^
G4E1^ab^	G1E4^ab^	G1E4^ab^	G2E1^b^	G1E1^a^	G2E1^ab^	G4E3^a^	G1E4^ab^	G3E3^ab^	G3E3^b^
G5E1^abc^	G1E3^ab^	G1E3^abc^	G4E1^c^	G4E1^ab^	G5E2^b^	G3E1^ab^	G3E3^b^	G2E1^ab^	G5E2^b^
G1E1^abcd^	G2E2^ab^	G5E1^abcd^	G1E1^d^	G1E3^abc^	G3E1^c^	G4E2^b^	G2E4^c^	G3E2^ab^	G5E1^bc^
G2E2^abcd^	G2E1^b^	G2E2^abcde^	G2E2^de^	G2E1^abc^	G4E1^c^	G3E3^bc^	G1E1^c^	G2E4^abc^	G3E4^bcd^
G4E2^bcde^	G3E3^b^	G3E3^abcde^	G3E1^e^	G3E1^abc^	G3E2^c^	G2E3^cd^	G5E1^cd^	G4E3^abc^	G4E4^cde^
G2E1^cdef^	G5E1^b^	G2E4^abcde^	G1E3^f^	G5E3^abc^	G2E2^d^	G2E1^d^	G2E3^cde^	G5E3^abc^	G2E2^def^
G3E3^def^	G5E3^b^	G2E1^bcde^	G5E2^fg^	G2E4^bc^	G1E1^d^	G4E1^de^	G5E3^cde^	G3E1^abc^	G4E3^defg^
G1E3^efg^	G5E4^b^	G5E3^bcdef^	G2E3^g^	G5E4^bc^	G1E3^d^	G5E1^ef^	G4E2^cde^	G5E1^abc^	G4E2^efgh^
G3E2^efg^	G2E3^b^	G3E1^cdef^	G4E2^h^	G5E2^cd^	G2E3^d^	G4E4^ef^	G5E2^cdef^	G2E2^abc^	G1E4^efghi^
G2E4^efg^	G1E2^b^	G1E2^cdef^	G3E3^h^	G1E4^cd^	G2E4^e^	G2E4^fg^	G3E4^cdefg^	G2E3^abc^	G2E4^efghi^
G3E4^fgh^	G3E1^b^	G2E3^cdef^	G2E4^h^	G2E2^cde^	G4E3^ef^	G5E2^gh^	G3E1^defg^	G4E1^abc^	G2E3^efghi^
G2E3^ghi^	G2E4^b^	G4E3^cdef^	G1E2^i^	G1E2^cde^	G5E3^efg^	G3E2^h^	G2E2^defg^	G3E4^abc^	G5E^efghi^
G1E2^ghi^	G3E4^b^	G3E4^cdef^	G3E4^i^	G4E4^cde^	G4E2^efg^	G3E4^h^	G4E3^defg^	G4E4^abc^	G4E1^fghi^
G5E2^ghi^	G4E4^b^	G4E1^cdef^	G4E3^i^	G4E3^def^	G3E3^fg^	G1E3^h^	G1E2^defg^	G1E3^bc^	G3E1^ghij^
G4E4^hi^	G5E2^b^	G4E4^cdef^	G1E4^i^	G3E4^efg^	G3E4^fg^	G1E2^i^	G5E4^efg^	G1E2^bc^	G5E4^hij^
G1E4^hij^	G3E2^b^	G5E2^def^	G3E2^i^	G2E3^fgh^	G1E4^gh^	G5E4^i^	G4E1^efgh^	G5E4^bc^	G3E2^ijk^
G5E3^ij^	G4E2^b^	G3E2^def^	G5E3^j^	G3E2^fgh^	G1E2^h^	G2E2^i^	G4E4^fgh^	G1E4^bcd^	G1E1^jk^
G4E3^ij^	G4E3^b^	G4E2^ef^	G5E4^j^	G4E2^gh^	G4E4^h^	G5E3i	G3E2^gh^	G5E2^cd^	G1E2^jk^
G5E4^j^	G4E1^b^	G5E4^f^	G4E4^j^	G3E3^h^	G5E4^h^	G1E4^j^	G1E3^h^	G4E2^d^	G1E3^k^

Traits are plant height (PH), number of leaves (NoL) and number of branches (NoB), leaf area (LA), fresh weight (FW), dry weight (DW), length of panicle (LP), weight of 1000 grains (P1000), and individual grain yield (IY) of the quinoa plant. Different lowercase letters within columns represent statistically significant differences (Least significant difference test, P < 0.05) where a is the largest value in each column. G1, G2, G3, G4, and G5 represent the Q1, Cahuil, G18, Isluga, andQ3 genotypes. E1, E2, E3, and E4 represent four environmental treatments, namely control, waterlogging, salinity, and drought stress, respectively.

**Fig 1:**
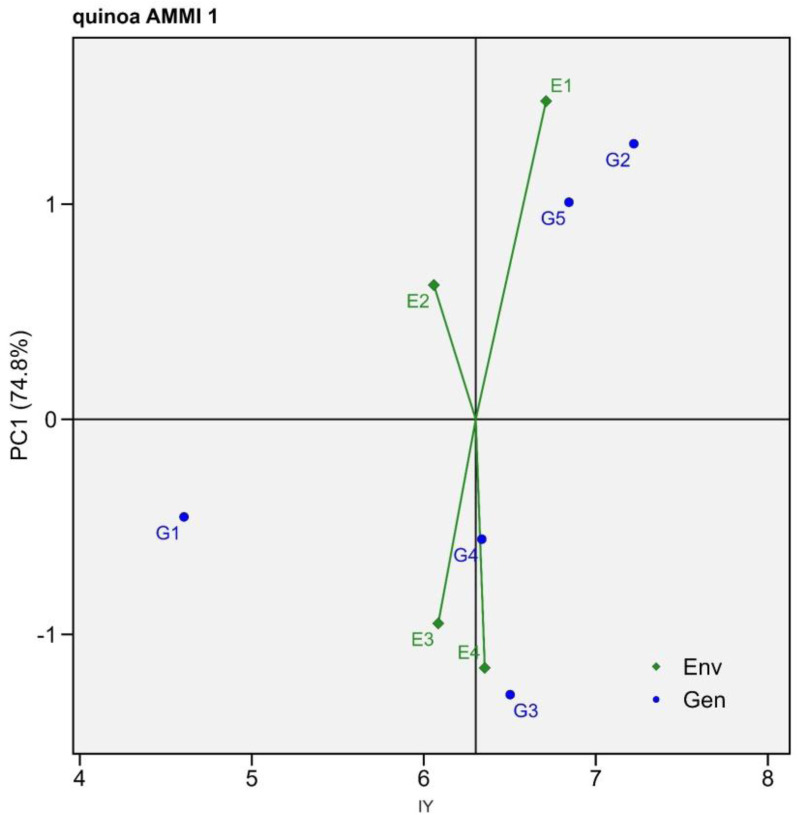
Additive main effects and multiplicative interaction (AMMI1) biplot based on Principal Component 1 (PC1), illustrating G × E interactions of quinoa genotypes. G1, G2, G3, G4, and G5 represent the Q1, Cahuil, G18, Isluga, and Q3 genotypes. E1, E2, E3, and E4 represent four environmental treatments, namely control, waterlogging, salinity, and drought stress, respectively.

The average individual grain yield of genotypes (IY) varied widely under environments, ranging from 3.77 g plant^-1^ (G1E3) to 10.29 g plant^-1^ (G2E1) (Table 2). To further investigate the G × E interactions for IY, the additive main effects and multiplicative interaction (AMMI) analysis was employed. The AMMI1 biplot, based on Principal Component 1 (PC1), is presented in Fig 1. This biplot illustrates the main effects of genotypes and environments along with their interaction effects on IY. PC1 was found to be highly significant, explaining 74.8% of the total variation (Fig 1.). Genotypes and environments positioned near the origin of the biplot show minimal interaction effects, suggesting stability across environments (i.e., broad adaptation). In contrast, those located farther from the origin exhibit greater interaction effects, indicating specific adaptation to certain environments.

**Fig 2:**
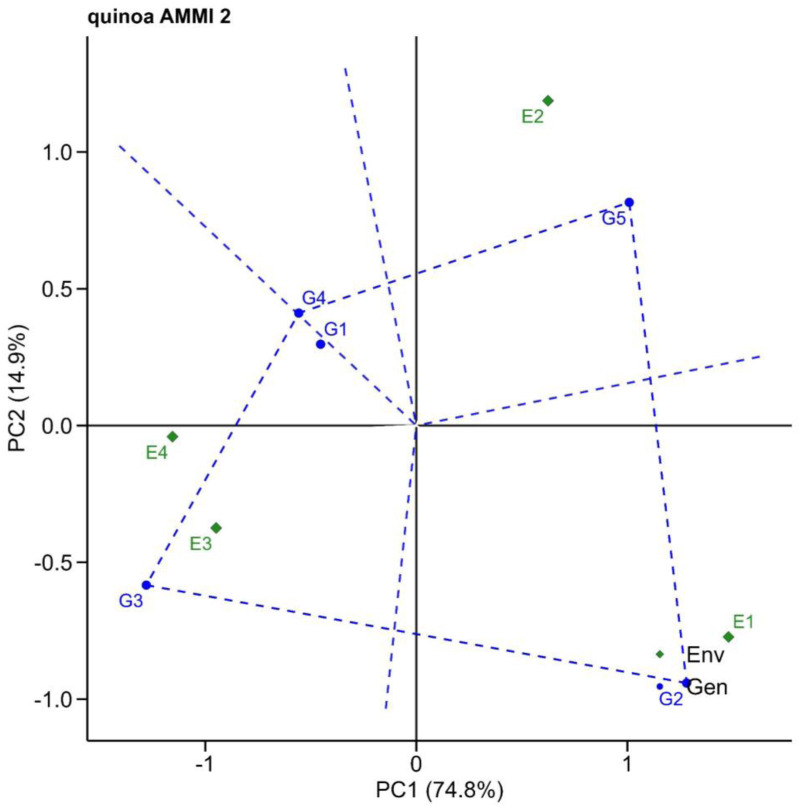
Additive main effects and multiplicative interaction 2 (AMMI2) biplots based on PC1 and PC2 illustrating G × E interactions of quinoa genotypes. G1, G2, G3, G4, and G5 represent the Q1, Cahuil, G18, Isluga, and Q3 genotypes. E1, E2, E3, and E4 represent four environmental treatments, namely control, waterlogging, salinity, and drought stress, respectively.

The AMMI2 biplot, based on PC1 and PC2, is shown in Fig 2. This biplot provides a more detailed visualization of the interaction patterns between genotypes and environments. It identifies which genotypes perform best under specific environmental conditions (specific adaptation) and which ones display broad adaptation. For instance, genotypes positioned close to a particular environment in the biplot are better adapted to that environment. The biplot analysis accounted for 89.7% of the total observed variation, with 74.8% explained by PC1, and 14.9% by PC2. Genotypes G3, G4, and G5 were situated at the corners, indicating their exceptional performance in specific environments. In particular, G3 shows good adaptability in environments E3 and E4 (indicating strong tolerance to drought and salinity), while G5 exhibits good tolerance in environment E2 (indicating strong resistance to waterlogging stress).

### Identification of ideal genotypes

**Fig 3:**
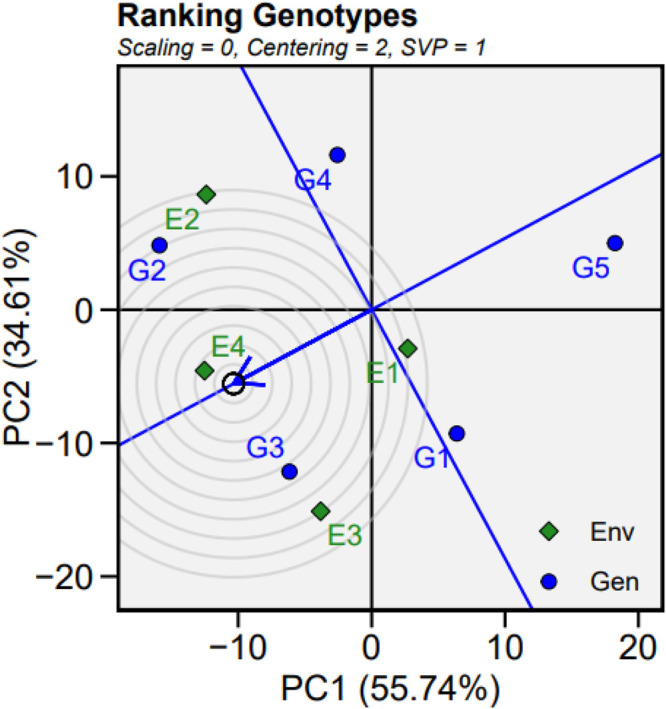
Genotype plus genotype by environment interactions (GGE) plot for genotype comparison with the ideal genotype plotted near the center of the concentric circles. G1, G2, G3, G4, and G5 represent the Q1, Cahuil, G18, Isluga, and Q3 genotypes. E1, E2, E3, and E4 represent four environmental treatments, namely control, waterlogging, salinity, and drought stress, respectively.

An ideotype or ‘ideal’ genotype is a model plant type designed to maximize yield in a specific environment through a combination of favorable traits. Assessing the performance of genotypes in relation to these ideotypes can guide prioritization of their use and breeding programmes. This ideal genotype will be characterized by high mean performance combined with stability across different environments (broad adaptation). A comparison of tested quinoa genotypes with such an ideal genotype is depicted in Fig 3, with the ideal genotype plotted at the center of concentric axis circles. Genotypes located closer to the ideal point are considered more desirable for agronomy or breeding purposes due to their consistent performance and adaptability. In this study, we found that some genotypes, notably G3 (G18) and G5 (Q3), were positioned closer to the ideal genotype, indicating their potential as stable performers across varying environmental conditions.

## Discussion

Although quinoa is recognized for its resilience to salinity and drought, our findings reveal that its growth and development can be significantly affected under these unfavorable water conditions. Indeed, drought stress in quinoa can considerably reduce physiological and morphological traits, including leaf area and dry matter accumulation [17,24,25]. Consistent with these studies, our results showed that salinity and drought negatively affected all measured traits, with reductions ranging from 4% to 59%, particularly causing serious decreases in LA and FW (Table 2). These results indicating that despite its stress-tolerant reputation, quinoa remains vulnerable to both abiotic stresses under extreme water imbalances, especially at sensitive stages such as flowering.

Drought stress (E4) had the second most severe decline in growth performance [26]. While E3 did result in reductions across all traits, it was less damaging than waterlogging. This finding is consistent with previous studies that report a significant reduction in dry biomass and photosynthetic capacity under limited water availability, but which note that quinoa often maintains plant height and continues reproductive development to some degree [17]. In our study, genotypes like G2 (Cahuil) and G5 (Q3) performed better under drought stress, exhibiting less severe reductions in traits like PH, NoB, and DW compared to other genotypes, although all genotypes still showed stress-induced reductions. This demonstrates that quinoa is sensitive during the vegetative stages, even in drought-tolerant genotypes.

Salinity stress (E3) while still negatively affecting growth, had the least impact of the three stress treatments, which is consistent with quinoa’s well-documented salt tolerance. Many quinoa varieties can maintain biomass production and physiological function at moderate to high salinity levels [17]. In our study, G2 (Cahuil) and G5 (Q3) performed relatively well under salinity stress, with fewer LA and DW reductions compared to waterlogging or drought conditions. However, salinity still resulted in significant differences from the control, especially in terms of dry weight, highlighting that, even in salt-tolerant species like quinoa, genotypic variability and environmental interactions play important roles in determining the degree of tolerance.

Like many upland crops, quinoa is highly sensitive to excess moisture [27]. While it is widely recognized for its drought tolerance, quinoa is considerably less adapted to prolonged hypoxic conditions caused by waterlogged soils [28,29]. This vulnerability has been documented in previous study with controlled growth chamber experiments, the altiplano variety ‘Sajama’ experienced marked declines in shoot and root dry weight, total chlorophyll content, alongside increases in soluble sugars and starch concentrations under waterlogged conditions [30]. Similarly, field trials in Brazil revealed that the variety ‘BRS Piabiru’ achieved optimal leaf development under moderate water availability, but excessive moisture led to reduced leaf traits, reinforcing the crop’s sensitivity to waterlogging stress [31]. The results of this study are consistent with previous findings, E2 has the most severe impact on both growth and yield in quinoa. The significant decline in SPAD values reflects inhibited photosynthetic capacity, which leads to reductions in NoL, NoB, LP, P1000, and overall IY. These results indicate that quinoa is particularly sensitive to waterlogging.

Although quinoa is known for its tolerance to drought and salinity [18], studies have shown that it is more tolerant to salinity than to drought [32]. Moreover, waterlogging has been demonstrated to cause significantly more severe effects than drought [33]. However, no study to date has comprehensively compared the impacts of these stress factors on the major growth and yield traits of quinoa. Consistent with previous reports, the present study revealed that among the four environmental treatments (waterlogging, drought, salinity and control) evaluated, waterlogging (E2) exerted the most severe negative effects on key agronomic traits of quinoa, including NoL, NoB, SPAD, LP, and especially IY. The order of stress severity was: Waterlogging (E2)> Drought stress (E4)> Salinity (E3)> Control (E1). These results highlight the particular vulnerability of quinoa to excess moisture, especially under tropical or subtropical conditions where heavy rainfall and poor soil drainage are common challenges.

The majority of potential quinoa cultivation zones in the Asian tropics, including Vietnam, are rainfed and, therefore, highly vulnerable to climate change-related water stresses [33]. In such contexts, the analysis of G × E interactions using robust statistical tools is essential for identifying high-performing and stable genotypes suitable for specific environments, in particular Vietnam and neighbouring countries. In this study, the AMMI and GGE biplot analyses revealed crossover interactions in genotype performance across stress environments, underscoring the necessity of evaluating cultivars under multiple stress conditions before recommending cultivation. Based on biplot ranking relative to the ideal genotype, G18 and Q3 demonstrated the highest performance and stability across environments, appearing near the center of the concentric circles. Our results suggest that G18 and Q3, alongside other tested genotypes such as Q1, Cahuil, and Isluga, show varying levels of adaptability under both drought and waterlogging, with G18 in particular showing consistent performance across environments. These genotypes can be considered for future multilocation field trials or as parental lines in breeding programs targeting the development of climate-resilient quinoa varieties for Vietnam and similar agroecological zones in the region [33].

Quinoa’s response to drought includes a variety of morphological, physiological, and molecular mechanisms, such as deeper rooting systems, stomatal regulation, antioxidant production, and abscisic acid (ABA) signaling [17]. In particular, ABA-mediated responses in drought-sensitive stages such as flowering have been documented in cultivars like ‘INIA-Illpa,’ ‘Titicaca,’ and ‘Achachino’ [17]. Such mechanisms can help explain the physiological adjustments observed under stress conditions in our study and should be further investigated in cultivars to strengthen breeding strategies for adverse abiotic environments.

## Conclusions

Our results demonstrated that genotype, environment, and their interaction (G × E) significantly affected all measured morphological and yield-related traits in quinoa at flowering stage (S1 Table). Substantial differences were observed among genotypes in their ability to adapt and perform under various abiotic stresses, including drought, salinity, and waterlogging. The AMMI analysis highlighted strong genotype-specific responses across environments, reinforcing the importance of multi-environment trials in identifying stable and high-performing genotypes. Among the evaluated genotypes, G18 and Q3 were closest to the ideal genotype in terms of both performance and stability of grain yield across environments, indicating their potential for breeding programs and cultivation in diverse agroecological zones. These findings provide a solid scientific foundation for the selection and development of climate-resilient quinoa varieties suitable for adverse growing conditions.

## Supporting information

S1 Table Raw data. (XLSX)
